# Familial Hemophagocytic Lymphohistiocytosis secondary to UNC13D mutation: a report of two cases

**DOI:** 10.1186/s12887-022-03746-9

**Published:** 2022-11-19

**Authors:** Payman Sadeghi, Golnaz Ghazizadeh Esslami, Hassan Rokni-Zadeh, Majid Changi-Ashtiani, Reihaneh Mohsenipour

**Affiliations:** 1 Children’s Medical Center, Pediatrics Center of Excellence, Tehran, Iran; 2 Department of Pediatrics, Tehran University of Medical Sciences, Tehran, Iran; 3 Pediatric Rheumatology Society of Iran, Tehran, Iran; 4 Department of Emergency, Children’s Medical Center, Pediatrics Center of Excellence, Tehran University of Medical Sciences, Tehran, Iran; 5 Departments of Newborn Nursery, Neonates and Pediatrics, Ziaeian Hospital, Tehran University of Medical Sciences, Tehran, Iran; 6 Department of Family Medicine, Ziaeian Hospital, Tehran University of Medical Sciences, Tehran, Iran; 7 Zanjan Pharmaceutical Biotechnology Research Center, Zanjan University of Medical Sciences, Zanjan, 45139-56184 Iran; 8 School of Mathematics, Institute for Research in Fundamental Sciences (IPM), Tehran, Iran; 9 Growth and Development Research Center, Children’s Medical Center, Tehran University of Medical Sciences, Tehran, Iran

**Keywords:** Case report, FHL (familial Hemophagocytic lymphohistiocytosis), HLH (Hemophagocytic lymphohistiocytosis), *UNC13D*

## Abstract

**Background:**

Hemophagocytic lymphohistiocytosis (HLH) is a life-threatening disease characterized by some clinical signs (e.g., non-remitting fever, hepatosplenomegaly) and laboratory findings (e.g., cytopenia, increased ferritin level, hypofibrinogenemia, lipid disorders, coagulopathy, and multiple organ failure). Depending on the etiology, HLH is divided into familial (i.e., primary) and acquired (i.e., secondary) forms. Familial HLH (FHL), an autosomal recessive condition, is classified into five subtypes based on underlying genetic defects. The *PRF1*, *STX11*, *UNC13D*, *HPLH1*, and *STXBP2* are the most well-known genes of this type which are related to granule-mediated cytotoxic T and Natural killer (NK) cells. The treatment is based on the HLH-2004 protocol.

**Case presentation:**

The current report presents two cases of HLH with presentations different from each other and previously reported cases. Case 1 was a 15-month-old boy with fever, skin rash, splenomegaly, and bicytopenia, raised triglyceride levels, AST (aspartate transaminase), and ALT (alanine aminotransferase), normal ferritin, and abundant hemophagocytic cell in bone marrow aspiration. He was diagnosed with HLH and received HLH protocol as treatment. The patient had a homozygous intronic mutation; NM_199242: c.2448-13G > A in *UNC13D*. The associated disease was Familial Hemophagocytic Lymphohistiocytosis 3 (FHL3). Case 2, a 37-day-old female presented with fever, a history of neonatal cholestasis, and huge hepatosplenomegaly. Her whole-exome sequencing report manifested that the patient had the same mutation as case 1. Unfortunately, both patients passed away.

**Conclusion:**

The sequencing of the entire *UNC13D* gene (coding and non-coding regions) is an applicable and valuable diagnostic procedure for the detection of deep intronic splicing variants and large inversions in patients with atypical manifestations of HLH (such as normal ferritin or triglyceride and cholesterol).

## Background

Hemophagocytic lymphohistiocytosis (HLH) is an aggressive and life-threatening disease that can be fatal [1]. It is characterized by some clinical signs (e.g., non-remitting fever, hepatosplenomegaly) and laboratory findings (e.g., cytopenia, increased ferritin level, hypofibrinogenemia, lipid disorders, such as hypertriglyceridemia, coagulopathy, and multiple organ failure) [2, 3]. Immune dysregulation in cytotoxic T cells, Natural killer (NK) cells, and histiocytes is considered the leading cause of this disease.

Depending on the etiology, HLH can be divided into familial (i.e., primary) and acquired (i.e., secondary) forms [4–6]. Familial HLH (FHL), an autosomal recessive condition, is classified into five subtypes based on underlying genetic defects. The *PRF1*, *STX11*, *UNC13D*, *HPLH1*, and *STXBP2* are the most well-known genes of this type.

Mutations associated with restrained cytotoxic responses of cytotoxic T and NK cells to B cells infected by Epstein-Barr virus (EBV) inducing X-linked lymphoproliferative syndrome (*SH2D1A*, *XIAP*) are the third reason. Nevertheless, other primary immunodeficiencies, including combined immunodeficiency, chronic granulomatous disease, autoinflammatory diseases, and antibody deficiencies, have been described to cause HLH [4–8]. Acquired or secondary HLH, also known as macrophage activation syndrome, mainly occurs after severe viral, bacterial, or fungal infections, metabolic disorders, malignancies, and rheumatologic and immune diseases, such as autoimmune or autoinflammatory diseases [4–6, 9–11].

The treatment is based on HLH-2004 protocol, including chemoimmunotherapy (corticosteroids, etoposide, cyclosporin A, and/or intrathecal methotrexate), supportive therapy, prophylactic antibiotics, and intravenous Ig (IVIG). Hematopoietic stem cell transplantation is recommended in selected patients with refractory and/or relapsed disease after appropriate chemoimmunotherapy [12–14]. This treatment is modified regarding the classification and pathophysiology of HLH.

T-cell-directed immunotherapy could be advantageous in patients with primary HLH. In contrast, strong immunosuppression therapy is contraindicated in patients with severe proceeding infections or some primary immunodeficiency diseases other than familial HLH and X-linked lymphoproliferative syndrome [3]. In addition, more T-cell targeting therapies, such as anti-thymocyte globulin, etoposide, and alemtuzumab, can be beneficial to patients with primary HLH due to overactivated T cells [4, 5].

The differentiation between the two mentioned types of HLH is sometimes tricky, particularly in the beginning; moreover, diagnosis at the right time is critical for initiating appropriate and indicated treatment. Therefore, we performed whole-exome sequencing on two separate patients with relatively different symptoms to find the same homozygous intronic *UNC13D* mutation.

## Case presentation

In this study, we present the case of two children with rather different clinical symptoms hospitalized almost at the same time in two different centers affiliated with Tehran University of Medical Sciences. Their ages, symptoms, and signs were different, especially at the beginning; nonetheless, whole-exome sequencing was employed due to the uncertainty surrounding the underlying etiology. Written informed consent was obtained from their parents.

### Case 1

The first case was a 15-month-old male infant born to consanguineous parents via normal vaginal delivery. He had normal growth and development until admission due to prolonged fever for 40 days with no specific pattern, vomiting, and maculopapular rash on his face. His physical examination revealed huge splenomegaly (diameter 120 mm) and fever. Liver function tests demonstrated a rise in aspartate aminotransferase (AST) of 88 IU/L and alanine aminotransferase (ALT) of 52 IU/L. His Complete blood count (CBC) showed bicytopenia (white blood cell count of 10.8 thousand/mm^3^, anemia with hemoglobin of 10.8 g/dl, and thrombocytopenia with a platelet count of 106 *103/μl). Triglyceride (TG) level was initially 425 mg/dl, which increased to 644 mg/dl. The cholesterol level was initially 86 mg/dl, which increased to 182 mg/dl. The c-reactive protein (CRP) was high (73 mg/dl) with a normal erythrocyte sedimentation rate (ESR) at the same time (14 mm/h). Remarkably, the value of serum ferritin was normal throughout all his disease period (150 μg/l).

His abdominal sonography illustrated splenomegaly with a diameter of 120 mm and homogenous echogenicity. Bone marrow aspiration (BMA) performed from the iliac crest appeared normal. He was diagnosed with macrophage activating syndrome in the context of systemic juvenile idiopathic arthritis by a consultant rheumatologist. He received intravenous methylprednisolone pulse therapy (30 mg/kg) and intravenous immune globulin (IVIG). He was discharged from the hospital with oral prednisolone at a dose of 1 mg/kg/day and readmitted with intermittent fever and vomiting after 45 days. He had bicytopenia, increased ALT and CRP during hospitalization, and his ferritin level was 248 μg/l. Antibody assessment against cytomegalovirus (CMV) and Epstein-Barr virus (EBV) displayed no abnormality.

The patient underwent BMA again, and bone marrow revealed abundant hemophagocytic cells. He was treated with cyclosporine A, etoposide, and dexamethasone according to HLH protocol 2004. After 1 month of treatment, CBC turned normal; CRP and TG decreased. Upon the completion of induction therapy with HLH protocol and initiation of maintenance therapy, the patient experienced a disease flare-up and was readmitted with fever and status epilepticus. Laboratory data revealed pancytopenia and increased levels of TG, AST, and ALT again; however, ferritin level was in the normal range, and brain computed tomography (CT) scan showed ventriculomegaly and Mega Cisterna Magna (Table 1).

**Table 1:** Timeline of clinical and laboratory presentation of case 1

	First admission	Second admission	Last admission
Clinical presentation	Fever, vomiting, skin rash, splenomegaly	Fever, vomiting, splenomegaly	Fever, status epilepticus
CBC	Bicytopenia	Bicytopenia	pancytopenia
ESR(mm/h)	14	21	16
CRP(mg/dl)	73 reduced to 4	46	5
AST(IU/L)	88	36	74
ALT(IU/L)	52	53	207
TG(mg/dl)	425 increased to 644	386	972
Chol(mg/dl)	86 increased to 182	127	
Ferritin(μg/l)	150	248	155
Bone marrow aspiration	Normal	hemophagocytic cells	

**Fig. 1:**
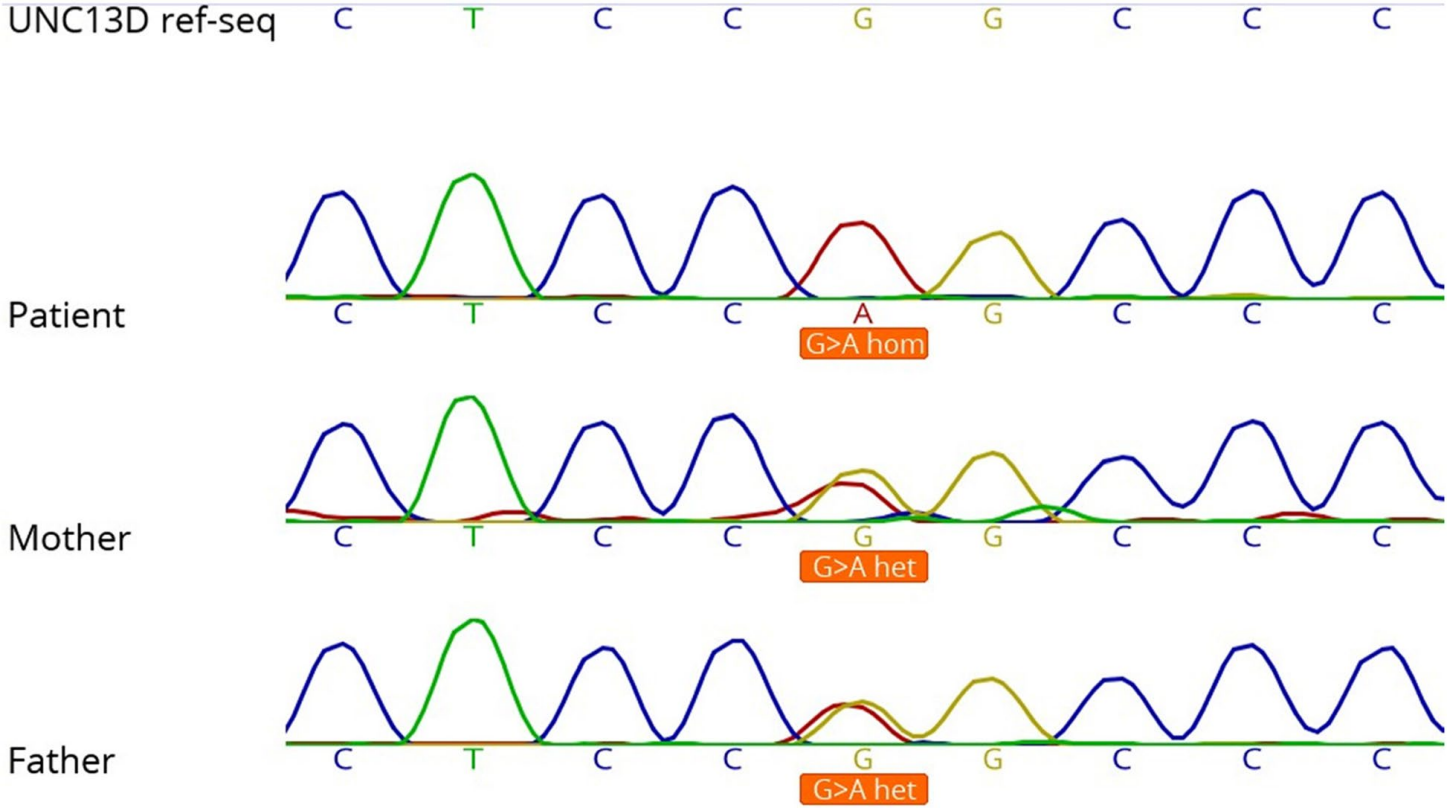
Sanger sequencing of c.2448-13G > A mutation in the studied families (Geneious software was used for sanger analysis)

In the last course of hospitalization, the patient passed away because of progressive brain involvement and hydrocephalus, and the whole-exome sequencing (on genomic DNA extracted from peripheral blood cells through an Agilent SureSelect V7 kit and Genome Analyzer HiSeq 4000 (Illumina, USA)) revealed a homozygous intronic *UNC13D* (NM_199242: c.2448-13G > A), probably affecting splicing. Although Sanger sequencing is not a routine survey for parents, since this mutation is transferred from parents or is a de novo mutation for the assessment of the next child, it was performed and confirmed that his parents were heterozygous carriers for this mutation (Fig. 1). The associated disease was Familial Hemophagocytic Lymphohistiocytosis 3(OMIM 608897) with autosomal recessive inheritance.

Furthermore, prenatal diagnosis with chorionic villus sampling was implemented in the 12th week of gestational age for the next child of this family and demonstrated that the fetus was only a heterozygous carrier of *UNC13D* mutation.

### Case 2

A 37-day-old female neonate born to consanguineous parents via cesarean section was admitted with fever (Temperature: 39 °C), cough, and a history of neonatal cholestasis. Her physical examination revealed fever and huge hepatosplenomegaly (with spleen preference). She had normal growth before admission despite the previous admission for cholestasis. Her liver function tests resulted in an AST of 33 IU/L that increased to 98 IU/L and an ALT of 21 IU/L that increased to 391 IU/L in her admission process. Her CBC revealed pancytopenia (leukopenia with white blood cell (WBC) of 3.13 thousand/mm^3^ and ANC of 210, anemia with hemoglobin of 5.1 g/dl, and thrombocytopenia with platelets of 41 *103/μl).

The TG and cholesterol levels were 113 mg/dl and 76 mg/dl, retrospectively. Ferritin level was increased to 2050 μg/l during her hospitalization. The CRP was 28 mg/L, and ESR was in the normal range (16 mm/h). Her abdominal sonography exhibited splenomegaly with a diameter of 144 mm and hepatomegaly with a diameter of 105 mm (both of homogenous echogenicity). The BMA and bone marrow biopsy were performed from the proximal tibia and appeared normal.

Her purified protein derivative (PPD) test turned negative, and a normal TORCH study (assessing antibodies against toxoplasmosis, rubella, CMV, and Herpes simplex virus [HSV]) was detected. The HIV ab was also negative. Metabolic screening tests, including MS/MS, ammonia, lactate, serum amino acid chromatography by HPLC, and urine reducing substance, all were normal. Nevertheless, the evaluation of Galactosemia, Gaucher’s disease, and Niemann-Pick’s disease were not feasible due to the previous history of blood transfusion. Furthermore, fibrinogen was in the normal range.

She was treated with prednisolone (4 mg/kg/day) and IVIG (2 g/kg Stat) as prescribed by a hematologist (with autoimmune thrombocytopenia and anemia diagnosis). Firstly, there was a rapid increase in platelet number and hemoglobin level. Nonetheless, the patient needed multiple blood transfusions and platelets due to sustained bicytopenia. Since the second case did not fulfill the criteria, she did not receive the 2004-HLH treatment protocol as suggested by a consultant rheumatologist. Finally, she died of severe sepsis by abdominal distention and positive blood culture with *Pseudomonas aeruginosa*. The whole-exome sequencing analysis detected a homozygous c.2448-13G > A *UNC13D* mutation, the same mutation observed in case 1. Sanger sequencing confirmed that his parents were also the heterozygous carriers of this mutation (data not shown). Sanger sequencing was performed since parents were young and desired to have a healthy child in the future.

## Discussion and conclusions

Familial hemophagocytic lymphohistiocytosis (FHL) is a challenging diagnosis due to the absence of any pathognomonic clinical and laboratory signs and symptoms. It is a rare autosomal recessive immune disease caused by the mutations of different genes associated with the formation and function of secretory lysosomes within cytotoxic T lymphocytes and NK cells. FHL type 3 (FHL3) accounts for approximately 30–40% of FHL, and mutation in the *UNC13D* gene, which encodes Munc13–4 protein, was reported as its underlying cause. This mutation results in defective cytotoxic granule exocytosis, followed by diminished cytotoxic activity of T lymphocytes [11].

In their study, Yoon et al. reported that FHL3 accounts for 89% of FHL cases in Korea, and nearly 20–25% in Japan, suggesting that *UNC13D* mutations may be responsible for a massive proportion of HLH patients in Asian countries [15]. Functional tests with and/or CTL cells, including perforin detection and degranulation assay quantifying CD107, a surface expression with flow cytometry, can direct genetic analyses [16].

The majority of mutations in the previously reported patients were discovered in deep intronic regions within intron 1 and 9, as well as an inversion in intron 30, advocating the fact that not only coding sequences of the *UNC13D* gene are essential for the assessment but also introns and non-coding regions should be sequenced and analyzed for the detection of deep intronic splicing variants and large inversions in patients who have failed to show mutations in whole-exome sequencing [17, 18]. The genetic study of both patients revealed a homozygotic intronic c.2448–13 G > A *UNC13D* mutation previously reported by Alsina et al. [19]. Compound heterozygous mutations reported in the *UNC13D* gene are frequent in FHL patients [20]. Nonetheless, this case study indicates that homozygous mutations are primarily observed in consanguineous families [17].

This case report confirms the literature claim that more than two-thirds of FHL3 patients suffer from fever and hepatosplenomegaly. Since our first case was presented with status epilepticus, neurological manifestations are also common among HLH patients, according to the literature. Liver failure, lymphadenopathy, consumptive coagulopathy, dermatologist manifestations, edema, and jaundice, like some other manifestations of the disease, were observed in our cases to some extent [18, 21]. Moreover, the laboratory findings of these two cases were in line with the literature (except for ferritin level in case 1), suggesting that neutropenia, thrombocytopenia, lack of NK cell activity, reduced fibrinogen, increased ferritin, and high level of triglycerides should raise doubts about the diagnosis of FHL3 [11].

The FHL3 mostly happens in the first year of life (with a median age of 3 months); nonetheless, the first case was older than 1 year [22]. Ferritin level has been reported high in most previous studies; however, it was interestingly normal in our first case, even during the active phases of the disease [23, 24]. A wide range of bacterial, viral, protozoal, and fungal infections are correlated with HLH [25]. Viral infections are supposed to provoke both primary and secondary types of HLH. Herpesviruses, particularly EBV, are the most prevalent viral infections [26]. Rickettsia [27], Mycobacterium [28], and Histoplasmosis [29] are the most frequent bacterial and fungal infections, retrospectively. Although it could not be justified, the sepsis induced by *Pseudomonas aeruginosa* could trigger the incidence of HLH in the second patient.

The solitary treatment for familial and severe HLH is bone marrow transplantation [13]. Based on the HLH 2004 protocol, an 8-week monotherapy utilizing dexamethasone, Cyclosporine A, or etoposide is considered the first-line treatment. However, the second case did not receive any treatment due to a failure to fill in the HLH 2004 protocol. Due to the involvement of the central nervous system in readmission, intrathecal methotrexate was administered to our first case with fever and status epilepticus, and bone marrow transplantation was considered the next step to put the disease in remission [30]; however, the neurological involvement did not respond well to the treatment, and after a course of status epilepticus, the patient’s condition got worse, and finally, he passed away.

The FHL, as a challenging diagnosis, may have a wide diversity of clinical manifestations and laboratory findings. Sequencing the entire *UNC13D* gene (coding and non-coding regions) is an applicable and valuable diagnostic procedure for detecting deep intronic splicing variants and large inversions in patients. Genetic assessment can significantly help in the event of HLH disease with atypical manifestation or situations when primary evaluations cannot detect etiology.

## Data Availability

Data of these two patients are available in the Children ‘s Medical Center hospital archive and can be public according to your request and The reported mutations in this study are submitted in ClinVar (Submission ID: SUB12176411).

## Abbreviations

HLH: Hemophagocytic lymphohistiocytosis
NK: Natural killer
FHL: Familial HLH
PRF1: Perforin 1
STX11: Syntaxin 11
UNC13D: Unc-13 Homolog D
HPLH1: Hemophagocytic lymphohistiocytosis 1
STXBP2: syntaxin binding protein 2
EBV: Epstein-Barr virus
IVIG: Intravenous immune globulin
PID: Primary immunodeficiency
WES: Whole exome sequencing
AST: Aspartate transaminase
ALT: Alanine Transaminase
CBC: Complete Blood Count
TG: Triglyceride
CRP: C-Reactive Protein
ESR: Erythrocyte Sedimentation Rate
BMA: Bone Marrow Aspiration
CMV: Cytomegalovirus
CT: Computed tomography
DNA: Deoxyribonucleic Acid
WBC: White Blood Cells
ANC: Absolute Neutrophil Count
BMB: Bone Marrow Biopsy
PPD: Purified protein derivative
TORCH: Toxoplasmosis, Others, Rubella, Cytomegalovirus, Herpes Simplex Virus
HSV: Herpes Simplex Virus
MS/MS: Tandem mass spectrometry
HPLC: High-performance liquid chromatography
FHL3: Familial HLH type 3
CTL: Cytotoxic T cell
CD 107: Cluster of differentiation, Cell markers in immunophenotyping
